# In Situ Investigation of the Iron Carbide Precipitation Process in a Fe-C-Mn-Si Q&P Steel

**DOI:** 10.3390/ma11071087

**Published:** 2018-06-26

**Authors:** Sébastien Y. P. Allain, Samy Aoued, Angéline Quintin-Poulon, Mohamed Gouné, Frédéric Danoix, Jean-Christophe Hell, Magali Bouzat, Michel Soler, Guillaume Geandier

**Affiliations:** 1 Institut Jean Lamour, UMR CNRS-Université de Lorraine 7198, 54000 Nancy, France; guillaume.geandier@univ-lorraine.fr; 2 Institut de Chimie de la Matière Condensée de Bordeaux, UPR CNRS 9048, 33608 Pessac, France; samy.aoued@gmail.com (S.A.); Angeline.Poulon@icmcb.cnrs.fr (A.Q.-P.); mm.goune@gmail.com (M.G.); 3 Normandie Université, UNIROUEN, INSA Rouen, CNRS, Groupe de Physique des Matériaux, 76000 Rouen, France; frederic.danoix@univ-rouen.fr; 4 Maizières Automotive Products, Arcelormittal Maizières Research SA, 57283 Maizières les Metz, France; jean-christophe.hell@arcelormittal.com (J.-C.H.); jean-christophe.hell@arcelormittal.com (M.B.); michel.soler@arcelormittal.com (M.S.)

**Keywords:** steel, Q&P, transition carbide, precipitation, HEXRD, TEM

## Abstract

Quenching and Partitioning (Q&P) steels are promising candidates for automotive applications because of their lightweight potential. Their properties depend on carbon enrichment in austenite which, in turn, is strongly influenced by carbide precipitation in martensite during quenching and partitioning treatment. In this paper, by coupling in situ High Energy X-Ray Diffraction (HEXRD) experiments and Transmission Electron Microscopy (TEM), we give some clarification regarding the precipitation process of iron carbides in martensite throughout the Q&P process. For the first time, precipitation kinetics was followed in real time. It was shown that precipitation starts during the reheating sequence for the steel studied. Surprisingly, the precipitated fraction remains stable all along the partitioning step at 400 °C. Furthermore, the analyses enable the conclusion that the iron carbides are most probably eta carbides. The presence of cementite was ruled out, while the presence of several epsilon carbides cannot be strictly excluded.

## 1. Introduction

Quenching and Partitioning (Q&P) is an annealing process proposed in 2003 to elaborate a new generation of advanced high strength steel (AHSS) for automotive construction [1,2]. The steels manufactured according to this new route show high yield strengths thanks to a refined microstructure as well as good ductility provided by the large fraction of austenite retained after processing, which enables a transformation induced plasticity (TRIP) effect. They are thus seen as possible solutions for automotive makers to lighten their body-in-white structures and to improve their crash resistance.

This processing route comprises an incomplete quenching step after an austenitic soaking in order to partially transform austenite into martensite followed by an isothermal partitioning step during which carbon is supposed to diffuse out from martensite (α’) to austenite (γ). This mechanism of retained austenite enrichment and stabilization is made possible if carbon does not remain trapped in the martensite. The final step consists generally in a rapid cooling during which a final martensitic transformation in stabilized austenite could occur. Usually, the Q&P heat treatment is thus defined by three key parameters, its quenching temperature (QT), its partitioning temperature (PT), and the partitioning time (Pt) [3].

The soaking conditions in the austenitic range have been chosen to suppress any trace of ferrite and to dissolve pre-existing cementite carbides. Ferrite is to be avoided as it causes a decrease in the yield strength of the steels. Residual carbides reduce the available carbon content to stabilize the retained austenite. Nevertheless, too severe austenitic soaking (too long or at too high temperature) leads to abnormal growth of austenite grains. Too large grains of austenite are unfavorable for the toughness of the steel and preclude in situ investigations using the considered diffraction setup (individual diffraction spots instead of Debye–Scherrer rings).

QT permits the initial fraction of martensite to set and thus the maximum fraction of austenite that can be stabilized during partitioning. If the initial fraction of martensite is high, the carbon content in martensite is sufficient to stabilize all the available austenite. On the contrary, if the fraction of martensite is low, the available carbon content in martensite is not sufficient to stabilize the austenite during the final quench. As suggested by the pioneering work of Speer et al. [1,2,3], the fraction of retained austenite can thus be maximized as a function of QT. PT and Pt serve to control the partitioning conditions, i.e., the diffusion of carbon from martensite to austenite. As the process is thermally activated, the higher the PT, the faster complete partitioning is achieved. As the partitioning mechanism is fast, the Pt parameter has in practice little effect except if other physical mechanisms take place. The so-called partitioning process can in fact be hindered by a possible carbide free bainitic transformation in retained austenite [3,4,5,6], by carbon segregations on martensite defects [7], and also by carbide precipitation in martensite [6,8,9,10,11]. These two last processes also occur commonly when tempering as-quenched martensitic steels [11,12]. In the Q&P treatment, the specificity is that they compete with carbon diffusion from martensite into retained austenite.

The main indirect effect of carbide precipitations is to trap carbon in martensite and thus block the enrichment of carbon in austenite. According to our knowledge, the direct effect of carbides on the mechanical properties of tempered martensite has not been demonstrated so far. Nevertheless, the direct and reliable measurement of the mass fraction of carbides is challenging in steels because of their low fractions and their nanometer sizes. Mossbauer spectroscopy or technique based on selective dissolutions allow accurate post mortem measures but carbide precipitation kinetics to our knowledge has never been determined in situ.

HajyAkbary et al. [6] reported the presence of epsilon (ε) carbides at the very early stage of the partitioning step in a Fe-0.3C-3.6Mn-1.6Si steel (in wt %). They claim that these transition carbides appear during the quenching step and then tend to dissolve slowly during partitioning step (PT = 400 °C). Their thermodynamic calculations exclude the occurrence of eta carbides. Pierce et al. [8,9] characterized by transmission electron microscopy (TEM) and Mössbauer spectroscopy (MS) the transition carbides in a Fe-0.4C-1.5Mn-1.5Si (in wt %) steel along different Q&P and Q&T (Quenching and Tempering) processes. In both cases, they report the precipitation of eta (η) carbides after tempering or partitioning at 400 °C. They also showed that eta carbides are essentially non-stoichiometric with compositions close to Fe_3_C, instead of Fe_2_C. This observation can explain why Toji et al. [11] identified their transition carbides as cementite (θ) particles, as they were only based on local composition measurement by atom probe tomography (APT). In addition, their observations of cementite are sustained by an original reassessed thermo-kinetic model. More recently, [9] Pierce et al. also observed cementite by TEM after Q&P at PT = 450 °C but attribute its occurrence to the decomposition of austenite at long partitioning time (Pt). Carbon clustering in martensite was also reported by Thomas et al. [7] in a highly alloyed system. In the literature, the nature, the composition of carbides, as well as their precipitation sequences thus all remain a bone of contention.

All these recent studies however lead to the conclusion that a certain fraction of carbon must also be trapped in martensitic laths, preventing a complete carbon partitioning between martensite and austenite, and thus limiting austenite enrichment. In a previous paper [13], we measured for instance that the carbon content in austenite of a Fe-0.3C-2.5Mn-1.5Si (in wt %) steel after quenching at 200 °C and 200 s partitioning at 400 °C was 1.05% (QT = 200 °C; PT = 400 °C; Pt = 200 s). As the final fraction of retained austenite is 12%, a simple mass balance shows that the overall residual carbon content in tempered martensite is 0.19%. The carbon depletion of martensite expected from the original theory of Speer et al. is thus far from being completed [3]. In addition, the lattice tetragonality of the martensite gives a mean carbon content of 0.09%C, meaning that 0.10% of carbon is segregated or precipitated in martensite. Even if the use of the body centered tetragonal (BCT) lattice of martensite is not considered as an absolute method, it shows that a large fraction of carbon is trapped in martensite. This value of 0.10% is consistent with the findings of Pierce et al. [8], which show that between 24% and 41% of bulk carbon content can be trapped in η carbides in their alloys.

The carbon balance was also sustained by the identification of a third phase on synchrotron X-ray diffractograms which can be indexed most probably as eta carbides according to the literature [13]. Nevertheless, their precise nature as well as their precipitation kinetics have not been analyzed and discussed so far. In the present paper, in the light of novel coupled SEM/TEM observations and diffraction experiments, we aim to confirm the nature of the third phase reported in our previous work. We show that particles observed by microscopy in tempered martensite are definitely transition carbides, most probably eta carbides but without excluding the presence of epsilon carbides. Based on these results, HEXRD experiments conducted on synchrotron beamlines was used to determine for the first time in situ the precipitation kinetics of the transition carbides throughout the studied Q&P schedule.

## 2. Materials and Methods 

The studied steel is a model alloy produced at laboratory scale, with composition Fe-0.3C-2.5Mn-1.5Si (in wt %). For more details about the manufacturing condition of the sample, please refer to [13]. This alloy is very close to the alloy studied by HajyAkbari et al. [6].

### 2.1. High Energy X-Ray Diffraction

High Energy X-Ray Diffraction (HEXRD) experiments conducted on synchrotron beamlines offer opportunities to follow in real-time the complex phase transformation processes and their possible interactions taking place in a thermomechanical treatment [4,13,14,15,16,17]. Our recent in situ experiments revealed for instance that intense second-order internal stresses at phase scale are produced all along the processing route of Q&P steels [13,14]. The stresses affect the way carbon enrichment in austenite during partitioning should be measured, and the apparent stability of retained austenite at room temperature. Such in situ experiments are thus the sole indirect method based on XRD to measure reliably carbon enrichment in austenite as they allow deconvoluting unambiguously the chemical and mechanical contribution in the austenite lattice parameter evolution.

**Figure 1:**
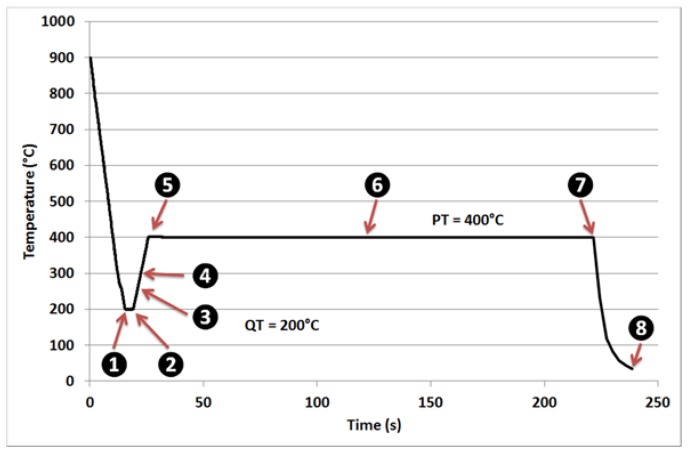
Quenching and Partitioning (Q&P) thermal treatment applied on the studied steel after a 5 min austenitization at 900 °C. The experiment was carried out in situ on a synchrotron beamline. 2D diffraction patterns were acquired every 0.1 s all along this cycle. Eight points of interest are highlighted.

The experiment analyzed in this paper was conducted at Deutsches Elektronen-Synchrotron (DESY, Hambourg, Germany). The experimental set-up is extensively described in our previous paper [13]. Petra P-07 beamline was operated at 100 keV using a powder diffraction configuration in transmission and a high throughput 2D detector which enables an acquisition rate higher than 10 Hz. The studied cylindrical sample (Φ = 4 mm; 10 mm height) was heated and cooled using a commercial Bähr dilatometer (TAinstruments, New Castle, DE, USA) available on the line. The in situ experiment was permitted to follow the phase transformations during a model Q&P schedule characterized by a quench temperature (QT) of 200 °C and a partitioning temperature (PT) of 400 °C. The detailed thermal schedule is represented in Figure 1. Different points of interest are reported in the cycle to simplify future discussions.

As shown in our previous papers [13,14], the low signal/noise ratio permitted by high energy enables the presence of transition carbides to be detected on diffraction spectra even at the beginning of partitioning (point 5 in Figure 1). These transition carbides were interpreted most probably as eta carbides on the sole basis of HEXRD results. In this paper, we aim to analyze and discuss further the nature of these carbides thanks to electron microcopy observations, and to appraise the precipitation kinetics.

### 2.2. Electron Microscopy

The HEXRD sample was also analyzed post mortem (state 8 in Figure 1) using conventional scanning electron microscopy (SEM) and TEM. By coupling these different techniques, it was possible to measure the size and the morphology of precipitates but above all to assess some additional information related to the nature of the carbides.

After a standard metallographic (polishing) preparation, the sample was etched with a combination of 1% Nital and Picral etchants for SEM observations using a JEOL 7001F Field Emission Gun (JEOL, Tokyo, Japan) operating at 10 kV. Thin foils were also prepared at low temperature using an EM-09100 JEOL cryo-ion slicer system (with a liquid nitrogen tank, JEOL, Tokyo, Japan). TEM observations were carried out on a JEOL 2100 (JEOL, Tokyo, Japan) operated at 200 kV.

## 3. Results and Discussions

### 3.1. Morphology and Size of Carbides by SEM Observations

**Figure 2:**
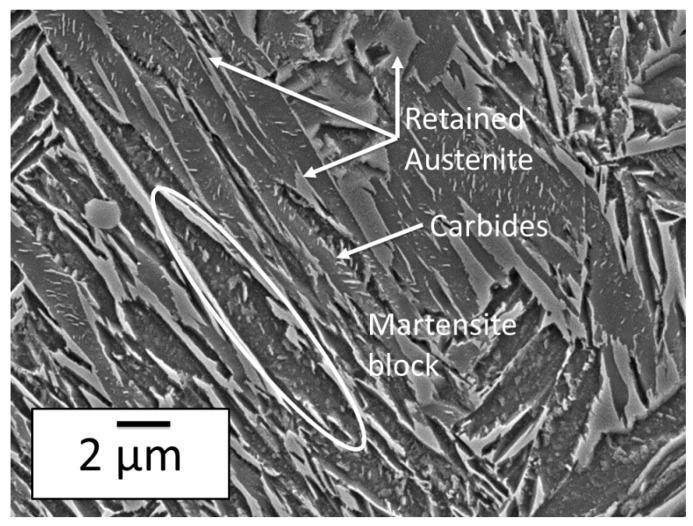
Scanning electron microscopy (SEM) micrograph of the studied steel after Q&P heat treatment (state 8). Etching has dissolved the tempered martensitic matrix preferentially. Intralath carbides and interlath retained austenite thus appear in clear contrast. A martensite block is also highlighted.

Figure 2 shows a SEM micrograph of the studied steel after Q&P heat treatment (state 8). The microstructure is obviously duplex with a fibrous martensitic matrix (dark contrast) containing interwoven fine austenite films and coarse austenite islands (clear contrast). According to our HEXRD measurements [13], the fraction of retained austenite is about 12% and the fraction of fresh martensite is lower than 1%. This latter fraction is determined during final cooling but the phase cannot be distinguished from austenite on the SEM micrograph. 

Tempered martensitic matrix contains also numerous intralath carbides which appear in bright contrast. Carbides show a platelet morphology. Their mean length is about 400 nm but the resolution of SEM does not permit a reliable estimate of their thickness (approx. a few nanometers).

### 3.2. Crystallographic Nature of Carbides by TEM Observations

**Figure 3:**
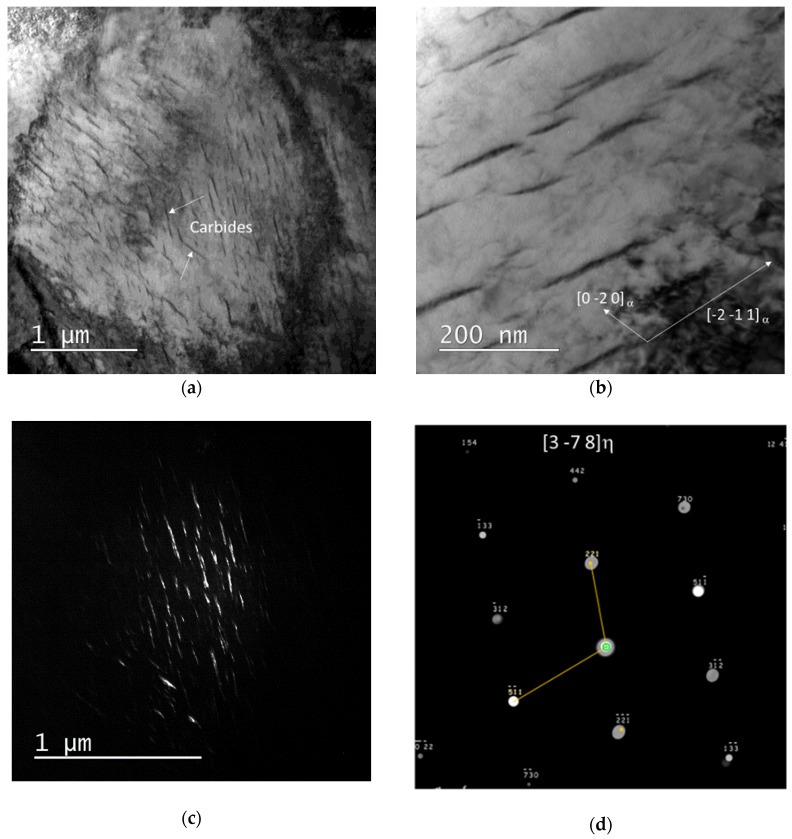
(**a**) Bright field transmission electron microscopy (BF TEM) micrograph of the studied steel after Q&P heat treatment (state 8). Arrows highlight the presence of 2 variants of carbides (dark contrast) in the tempered martensitic matrix (wide block). (**b**) BF TEM micrograph of the studied carbides aligned along martensite [-2-11]_α’_ (zone axis [210]_α’_). (**c**) Dark field (DF) image using (111)_η_ spot. (**d**) Indexed diffraction pattern (zone axis [3-78]_η_) of the η carbides aligned along martensite [-2-11]_α’_.

TEM observations were conducted first to confirm SEM observations regarding the morphology and the size of precipitates, but also to investigate their crystallographic structure. Figure 3a shows a bright field (BF) micrograph obtained on a wide block of tempered martensite. In that case, two variants of carbides were observed. In the literature, the majority of the linear precipitates are reported to align along martensite [010] and [001] crystallographic directions [6,18].

According to the literature, three types of carbides have been reported in Q&P steels: θ, ε, and η. The crystallographic structures of ε and η carbides are very close (η carbide structure is in fact pseudo-hexagonal and similar to the hexagonal ε carbide one) and are often difficult to distinguish from TEM experiments, especially when carbides are non-stoichiometric, and thus with vanishing super-lattice diffraction spots [8,17]. In this study, to determine the type of carbides (θ, ε, or η), we chose to work with those aligned along martensite [-2-11]_α’_ (Figure 3b). Selected area electron diffraction (SAED) observations were conducted in the same area with different zone axes for carbides. It was possible to interpret unambiguously all diffraction patterns using an orthorhombic structure and with lattice parameters consistent with the η structure. Figure 3c shows a dark field (DF) image using the (111)_η_ reflexion of the SAED pattern. Figure 3d shows for instance, a diffraction pattern of the carbides observable in Figure 3b and indexed considering the η carbide structure (Pnnm). This last zone axis is specific of the η structure.

The presence of the highly distorted and magnetic ferritic matrix make it difficult to obtain clear diffraction conditions, which jeopardize the possibility to confirm for certain the nature of all the precipitates using TEM, and in particular to exclude the presence of epsilon carbides. Nevertheless, the presence of cementite even at lath boundaries was ruled out.

The direct observation of samples by SEM and TEM after heat treatment shows a uniform distribution of carbides with platelet morphology (about 400 nm wide and 5 nm thick). The resulting microstructure is thus very similar to those reported by Pierce et al. [8,10] and by HajyAkbary et al. [6]. The techniques used do not permit however a reliable density of precipitate to be measured and thus a correct estimate of the carbide fraction.

### 3.3. Crystallographic Nature of Carbides by HEXRD

**Figure 4:**
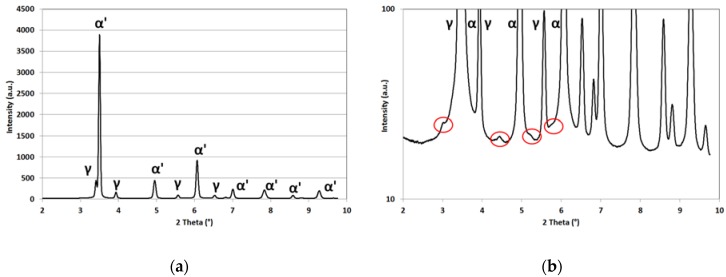
(**a**) 1D full scale diffractogram after circular integration of 2D Debye–Scherrer pattern (state 8 at room temperature after Q&P treatment). Major diffraction peaks are indexed and attributed to austenite (γ) and martensite (α’) respectively. (**b**) Enlargement of (**a**) (log scale) reveals the presence of minor diffraction peaks in between major peaks’ shoulders (red circles). These minor diffraction peaks are attributed to carbides.

All 2D X-ray diffraction patterns obtained all along the heat-treatment (one experimental pattern every 0.1 s as shown in Figure 1) were integrated circularly to obtain 1D diffractogram [19]. Such a procedure permits in particular statistical fluctuations and texture effects to be minimized, as discussed in [14]. Figure 4a shows an example of such a 1D diffractogram obtained in final state 8 (full intensity scale). The two families of diffraction peaks from the major constituents (martensite and austenite) are highlighted. Austenite (γ) diffraction peaks can be modelled considering a fcc lattice (space group Fm-3m) and martensite (α’) peaks considering a bct lattice (space group I4/mmm) as explained in our previous work [13,17]. The fraction of bainite formed during the considered heat treatment is low (less than 3%). This latter fraction was measured by HEXRD considering that the increase in bct phase fraction during partitioning is solely due to the bainitic transformation (no interface mobility between martensite and austenite).

The presence of carbides can only be revealed by enlarging the scale and by examining carefully the shoulders of the main peaks. Figure 4b evidences minor diffraction peaks emerging significantly from the statistical fluctuations (highlighted by a red circle) using a log scale. These diffraction peaks are not possible to observe with a conventional laboratory set-up from the author’s knowledge.

The similarities of η and ε crystallographic structures raise the same issues as in TEM when identifying these additional diffraction peaks. X-ray diffraction (XRD) patterns of the three possible structures were simulated using Fullprof [20] assuming their compositions and crystallographic structures. Table 1 shows the space groups, lattice parameters, and compositions used for the calculations. Other values can be found in the literature for the lattice parameters [6,21] but the differences are minor and do not affect the conclusions.

**Figure 5:**
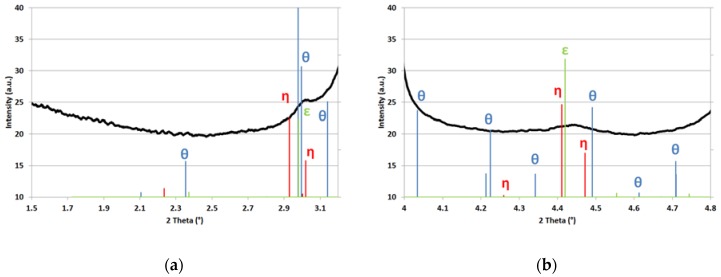
1D experimental diffractogram (black continuous curve) and position of simulated diffraction peaks of possible carbides (cementite in blue, eta carbide in red, and epsilon carbide in green); (**a**,**b**) diffraction angle windows corresponding to the first and second minor diffraction peaks in Figure 4b respectively.

Figure 5a,b show the expected positions of diffraction peaks of the three studied structures in the range of the two first minor diffraction peaks highlighted in Figure 4b. For each given structure, the relative heights of the peaks were calculated using structure and Lorentz polarization factors, and thus can be compared. Nevertheless, as the maximum intensities for each structure were chosen arbitrarily, it is useless to compare their absolute heights. θ appears in blue, η carbide in red, and ε carbide in green respectively. 

All minor diffraction peaks observed in Figure 4b can thus be accounted for either by η or by ε structures. On the contrary, cementite cannot explain in particular the wide peak observed at 4.45° and would have produced extra peaks at 4.22° and at 2.35° which are obviously not visible. Hence, as by TEM, the presence of cementite can be ruled out within the resolution limit of the technique (typically 0.1% mass fraction if the size of precipitates is sufficient).

Nevertheless, it is not possible to conclude with confidence on the nature of the observed transition carbides using our HEXRD set-up. When comparing expected diffractograms of η and ε, the sole discriminative and isolated fundamental peak is the (002)_η_ expected at 5.02°. Unfortunately, if this particular peak exists, it is convoluted with a major peak of the diffraction pattern associated to martensite and thus undetectable. Nevertheless, the width and the asymmetry of the minor diffraction peaks observed at 3.00° and 4.45° plead in favor of an η structure as suggested by Pierce et al. [8,9,10]. This result is also sustained by our own TEM observations. 

### 3.4. Precipitation Kinetics

Whatever the considered structure (η or ε), the mass fraction of transition carbide was estimated around 0.45% using a Rietveld simulation of peak height (at the end of the thermal treatment, i.e., at point of interest 8). Even if our in situ diffraction set-up does not permit with certainty the nature of the transition carbide to be confirmed, it enables following of the carbide precipitation sequence.

**Figure 6:**
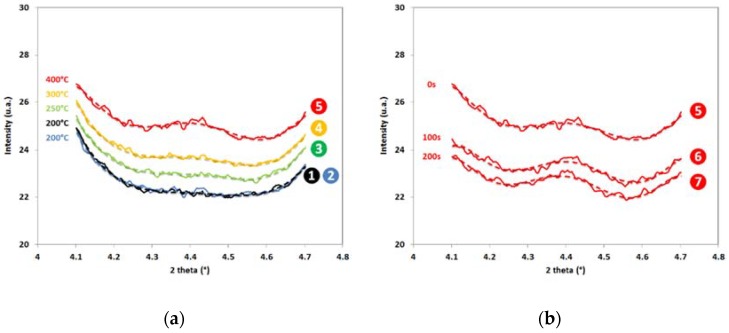
Experimental 1D diffractograms in a reduced angular windows corresponding to the second minor diffraction peak in Figure 4b obtained at different steps of Q&P treatment (**a**) points 1 to 5 according to Figure 1, i.e., after quenching and during reheating (**b**) points 5 to 7 according to Figure 1, i.e., during partitioning.

Figure 6a,b shows the evolution of the experimental diffraction patterns in the angular window corresponding to the distinctive diffraction peak of transition carbides at 4.45°. They are plotted for the points of interest indicated in Figure 1 and correspond respectively to the quenching step (1 and 2), the reheating step (3, 4, and 5) and the partitioning step (5, 6, and 7). The color code refers to the temperature. For the sake of readability, a 6th order polynomial fit has been adjusted on each experimental curve to highlight the main variations out from the statistical fluctuations.

At high and low diffraction angles in the studied range; the increases in intensity correspond to the shoulders of main diffraction peaks, to the (200)_γ_ peak on the left, and to the (200)_α’_ peak on the right. In between, the minor diffraction peaks appears already at 250 °C (point 3) as a small deviation from the flat signal observed after quenching (no evolution between points 1 and 2). The hump becomes more and more pronounced during reheating (between points 3 and 5). The mean intensity of the diffraction pattern increases during the reheating sequence because of the Debye–Waller overall isotropic displacement. During partitioning, the mean intensity decreases probably owing to a recovery process in major phases which leads to a narrowing of diffraction peaks and shoulders. Nevertheless, during this stage (points 5 to 7) at constant temperature, the relative height of the minor peak does not evolve significantly.

It is difficult to quantify with a reasonable precision the fraction of transition carbides all along the partitioning step, but our experiment shows undoubtedly that precipitation of carbide starts between 200 °C and 250 °C in the studied case, i.e., at the very beginning of the reheating step even if the reheating rate is high (50 °C/s). The comparison between patterns at points 1 and 2 shows no evolution during the 5 s pause before reheating at QT. This absence of evolution is the proof that transition carbide precipitation does not start during the quenching step after austenitization for the studied cooling rate, contrary to the post mortem observations of HajyAkbary et al. [6] or Pierce et al. [9] at room temperature.

This precipitation occurs even before the carbon enrichment process in austenite estimated at 270 °C for the studied case [13]. During partitioning, the fraction of transition carbides seems to remain constant.

## 4. Conclusions

As a conclusion, the experiments presented in this paper uphold the carbon mass balance established by HEXRD in our previous papers to explain the carbon enrichment in austenite after Q&P in a Fe-0.3C-2.5Mn-1.5Si steel [13]. Post mortem SEM and TEM observations after the heat treatment revealed the presence of numerous carbides in martensite, which prevents a complete partitioning of carbon into austenite in the studied conditions (QT = 200 °C, PT = 400 °C, Pt = 200 s). Carbon content in austenite (1.05%) is thus far lower than the composition expected from the sole constrained para-equilibrium (CPE) condition (about 1.9%) [3]. These carbides have a platelet morphology with a mean diameter of 400 nm and a thickness of approx. 5 nm. TEM observations ruled out the presence of cementite in the studied condition (even at martensite lath boundaries) but were not able to determine unambiguously the nature of the observed transition carbide (η or ε).

Our synchrotron experiments also permitted investigation for the first time the nature of carbides in Q&P steels by XRD. Diffraction patterns revealed the presence of a third phase in addition to austenite and martensite, which can be indexed as η or ε carbides. Despite a very low noise/signal ratio, it was not possible to conclude on the nature of the transition carbides. The asymmetry of minor diffraction peaks however pleads in favor of the η structure, as claimed by Pierce et al. [8,9,10]. No cementite was detected on the contrary with a high level of certainty, supporting the TEM observations.

As the experiment was carried out in situ with a high acquisition rate, real time precipitation kinetics was followed. Contrary to HajyAkbary et al. [6] and Pierce et al. [8,9,10], it was observed that transition carbide precipitation does not start during quenching, but during reheating below 250 °C, before carbon enrichment in austenite at 270 °C. In the studied conditions, the maximum fraction of precipitate is reached at the beginning of partitioning (about 0.5%). During the partitioning step (200 s at 400 °C), the fraction of precipitate does not evolve significantly (no dissolution), as already observed by Pierce et al. [8].

## Figures and Tables

**Table 1:** Space groups and lattice parameters of carbides used for X-ray diffraction (XRD) pattern simulations with Fullprof.

Carbide	Space Group	a (nm)	b (nm)	c (nm)	Composition	Ref.
θ	Pnma	5.0696	6.7671	4.5159	Fe_3_C	[22]
η	Pnnm	4.7040	4.3100	2.8300	Fe_2_C	[23]
ε	P6_3_22	4.7670	4.7670	4.3540	Fe_3_C	[24]
